# Effect of panretinal photocoagulation on optic nerve head blood flow with OCT angiography in patients with diabetic retinopathy

**DOI:** 10.12669/pjms.36.5.2190

**Published:** 2020

**Authors:** Saher Akbar Amanat, Asad Aslam Khan, Haroon Tayyab, Sohail Sarwar

**Affiliations:** 1 Saher Akbar Amanat, FCPS. Vitreoretina Fellow, Eye Unit III, Mayo Hospital, Lahore, Pakistan; 2 Asad Aslam Khan, MS Ophthalmology, FACPS, FCPS Ophthalmology, PhD. Fellowship in Paediatric Ophthalmology and Strabismus, Eye Unit III, Mayo Hospital, Lahore, Pakistan; 3 Haroon Tayyab, FCPS (VR), FICO Fellowship Vitreoretina (Japan). Eye Unit III, Mayo Hospital, Lahore, Pakistan; 4 Sohail Sarwar, MS Ophthalmology, PhD. Eye Unit III, Mayo Hospital, Lahore, Pakistan

**Keywords:** Proliferative Diabetic Retinopathy, Panretinal Photocoagulation, Optic Nerve Head Blood Flow, OCT Angiography

## Abstract

**Objective:**

To investigate the effect of pan retinal photocoagulation (PRP) on Optic nerve head blood flow with OCT angiography in patients with proliferative diabetic retinopathy.

**Methods:**

This prospective interventional study was conducted at Eye Unit III, Institute of Ophthalmology, Mayo Hospital, Lahore, over a period of seven months i.e. from 10th May 2019 to 10^th^ November 2019. Thirty-five patients having proliferative diabetic retinopathy were included in this study. Ocular blood flow was measured with OCT Angiography, then pan retinal photocoagulation was performed and patients were called for follow-up after one month and ocular blood flow was measured again with OCT Angiography. The difference in the blood flow was calculated. Frequencies and percentages were calculated for the categorical data and mean and standard deviations were calculated for the continuous data. Wilcoxon matched-pairs signed rank test was performed and effects of PRP on blood flow were compared. Significance level was taken as p≤0.05.

**Results:**

Out of 35 patients, 30 patients (85.71%) had decreased post PRP blood flow, four patients (11.43%) had increased post PRP blood flow and one patient (2.86%) did not have any effect.

**Conclusion:**

OCT angiography revealed there is significantly reduced optic nerve head blood flow in PRP treated eyes compared to non-PRP treated eyes.

## INTRODUCTION

Diabetic retinopathy is one of the important and leading causes of visual loss among adults. It is among the main complications of uncontrolled diabetes, others being diabetic neuropathy and diabetic nephropathy.^1^,^2^ In Pakistan, the prevalence of diabetes mellitus is increasing day by day and is estimated to be around 6.9% among adults and is expected to double by 2040.^3^ This number is observed to be 8.8% in Indian population.^4^ Majority of the patients with uncontrolled diabetes have to deal with retinopathy and macular edema at some stage of their disease. There are many treatment choices available which can be used to symptomatically treat the patients of diabetic retinopathy, however the present-day understanding of these options is limited and does not provide options for rescue strategies and complete treatment.^5^

Diabetic retinopathy is due to the retinal damage, specifically to its small blood vessels, caused by increased blood glucose levels. It may be proliferative diabetic retinopathy (PDR) or non-proliferative diabetic retinopathy (NPDR). New blood vessels develop in the retina and are the most severe manifestation of retinopathy because these vessels are abnormal in structure. The process of development of new blood vessels is known as neovascularization and is a hallmark of proliferative retinopathy. New blood vessels tend to bleed easily and can result in vitreous hemorrhage.^6^ Later on, a scar develops and contracts, leading to retinal detachment.

According to the studies, PRP helps to improve the oxygen supply of ischemic inner retinal layers. It damages exceedingly active photoreceptor cells thus improving the oxygen flow to the retinal layers. Choroid circulation might be affected by impairment of outer retinal tissue and pigment epithelium.^7^ It has been suggested that PRP can improve the oxygen supply to the inner ischemic layer of retina by destroying cells which have high metabolic activity. This mechanism has been observed in some of the animal studies.^8^

We searched for this topic on PubMed, EMBASE, Cochrane library, and Google Scholar but found no significant publications on the effect of PRP on optic nerve head blood flow in patients having diabetic retinopathy using OCT Angiography. This study was conducted in order to explore the effect of panretinal photocoagulation on optic nerve head (ONH) blood flow by measuring the capillary density rather than loss of ganglion cell in nerve fiber layer.

## METHODS

This prospective interventional study was conducted in Eye Unit III, Institute of Ophthalmology, Mayo Hospital Lahore for the period over a period of seven months i.e. from 10th May 2019 to 10^th^ November 2019. Thirty-five patients presenting with proliferative diabetic retinopathy and having age of 45-65 years were included in this study through non-probability purposive sampling. Permission from the hospital ethical committee (No.707/RC/KEMU, Date: 02-05-2019) was sought before commencement of the study.

Patients with proliferative diabetic retinopathy (due to type 1 or 2 diabetes mellitus), intraocular pressure < 18 mmHg, non-glaucomatous optic disc characteristics at fundus examination, vertical cup-to-disc (C/D) ratio <0.7 and absence of media opacities were included in the study. Patients presenting with history of incisional surgery in investigational eye, anti-VEGF therapy, use of steroids for diabetic macular edema at least 3 months, presence of macular abnormality, for example, asymptomatic pigment epithelial detachment or choroidal neovascularization were excluded from this study.

After taking informed consent, socio-demographic data including age, gender, address, phone number, occupation and level of education were noted on a prescribed form. OCT Angiography was performed before applying double-frequency Nd: YAG laser using machine Optovue RTVue XR Version 2017.1.0.15. Double-frequency Nd: YAG laser was used for treatment. For each session, photocoagulation was performed with 400-micron spot sizes with pulse duration of 0.15 seconds and in a single session, 3500 to 4000 shots were given to complete PRP. At the end of one month, follow up of the all patients was done. Intraocular pressure was measured with Goldmann applanation tonometer along with the fundus examination and OCT angiography.

OCT Angiorgraphy is a non invasive technique to detect the moving erythrocytes inside the vasculature of the retina. The structures in the retina are static, the only changes are caused by blood flowing through vasculature. After correcting for gross eye motion (by using motion tracking or software processing), the only differences remaining between OCT images of the same point in the eye would represent blood flow. The image-processing software was developed to measure optic nerve blood flow by measuring capillary density on the whole disc. Capillary density was measured in percentages before and after applying the laser. The number of visits and the number of burns needed to complete the initial treatment was also recorded. The need for additional laser treatment was decided by the clinical presentation. If the lesions persisted, further scatter photocoagulation was done. In cases of non-resolving vitreous hemorrhage affecting the vision or a traction retinal detachment, the patients were referred for vitrectomy and endolaser photocoagulation. A circle was set around the optic nerve head in order to calculate the changes in optic nerve head blood flow and measurement of capillary density was done twice.

All the data was entered on SPSS version 23. Frequencies and percentages were calculated for the categorical data such as gender and patients showing effect of PRP on blood flow. Mean and standard deviations were calculated for the continuous data such as age, pre PRP blood flow, post PRP blood flow and difference in the blood flow. Wilcoxon matched-pairs signed rank test was performed and effects of PRP on blood flow were compared. Significance level was taken as p≤0.05.

## RESULTS

Thirty-five patients underwent pan retinal photocoagulation over a period of seven months i.e. from 10th May 2019 to 10^th^ November 2019 at Eye Unit III, Institute of Ophthalmology, Mayo Hospital Lahore. There were 20 (57.1%) males and 15 (42.9%) females with a minimum age of 45 years, maximum age of 65 years and a mean age of 55.26 ± 5.57 years. Pre PRP blood flow was having a minimum value of 35.80%, maximum of 56.80% and a mean of 45.84 ± 5.05. Post PRP blood flow was having a minimum value of 28.70%, maximum of 52.50% and a mean of 40.05 ± 5.36. The difference between pre and post-PRP blood flow was also measured. It was having a minimum value of -4.00%, the maximum value of 20.40% and a mean of 6.11 ± 5.04%. Table-I.

**Table-I:** Demographic and pre and post Panretinal photocoagulation blood flow data.

Parameter	Minimum	Maximum	Mean ± SD.
Age (years)	45	65	55.26 ± 5.57
Pre PRP Blood Flow (%)	35.80	56.80	45.84 ± 5.05
Post PRP Blood Flow (%) (After one month of PRP)	28.70	52.50	40.05 ± 5.36
Difference (Pre – Post) (%)	-4.00	20.40	6.11 ± 5.04

Gender	*Male*	*Female*	*M:F Ratio*

	20 (57.1%)	15 (42.9%)	4 : 3

Wilcoxon matched-pairs signed rank test was performed (p <0.001). Out of thirty-five patients, thirty patients (85.71%) have decreased post PRP blood flow, 04 patients (11.43%) have increased post PRP blood flow and 01 patient (2.86%) did not have any effect. The difference in the number of patients showing different type of responses to PRP was significant (p<0.001). Table-II.

**Table-II:** Male and female distribution and the effect of PRP on blood flow.

	Dec. blood flow	Inc. blood flow	No effect	Total	
*Male*	17 (48.57%)	2 (5.71%)	1 (2.86%)	20 (57.14%)	(p <0.001)
Female	13 (37.15%)	2 (5.71%)	0	15 (42.86%)	

Total	30 (85.72%)	4 (11.42%)	1 (2.86%)	35 (100%)	

## DISCUSSION

In this study, blood flow in optic nerve head (ONH) was measured before and after one month of the treatment in 35 patients of diabetic retinopathy. Our study revealed that 30 patients (85.72%) had decreased blood flow of the optic nerve head after one month of the procedure (p < 0.001). Choroidal thickness is changed in diabetic retinopathy and affected by the extent of disease.^9^ Iwase et al.^7^ also suggested from their study that retinal blood flow was significantly decreases in the eyes which were treated with PRP as compared to normal eyes or those which were not treated. Similar results were also observed by Song Y et al.^10^ They observed that there was a maximum difference of 20.40ml/min in the RBF following panretinal photocoagulation in the patients if diabetic retinopathy. These results show that PRP is effective treatment provided diabetic control is maintained.

Zhu et al.^11^ conducted a study on diabetic patients suffering from early proliferative neuropathy and severe non-proliferative neuropathy. They observed a significant decrease in the choroidal thickness of the coagulated area following PRP. These effects reflect the redistribution of choroidal blood flow critical in maintaining retinal metabolism. Zhang et al.^12^ conducted a study in 2015 on 32 patients. They performed PRP and followed the patients for three months. They observed decrease in the choroidal thickness which indicates that PRP either causes atrophy of choroidal vessels or decrease the vascular permeability within choroid, over a time period of three months.

In current study, four patients (11.42%) had increased retinal blood flow after one month of PRP. Two females and two males having ages less than 60 years fall in this category. Possible explanations for this variety are unidentifiable. Kang et al.^13^ also reported similar findings in their study.

Okamoto et al.^14^ observed that subfoveal choroidal thickness was 327.4µm before performing PRP. This thickness decreased to 286.3µm after 01 month, and to 285.0 µm after 03 months of performing PRP. The correlation between subfoveal thickness choroid and blood flow in the same area was observed to be significant. Kim et al.^15^ have explained that the thickness of choroid increases gradually as the extent of disease progresses from normal to mild, moderate, and severe nonproliferative diabetic retinopathy and then to proliferative retinopathy. However, this choroidal thickness was observed to decrease in the eyes which had been treated with PRP.

Another study was conducted by Yamada et al.^16^ and they used pattern scan laser for PRP treatment. They observed reduced retinal blood flow after the treatment. They suggested that measuring retinal blood flow is an effective method to evaluate the efficacy of PRP treatment or extent of proliferative diabetic retinopathy. Lee et al.^17^ witnessed in their study that venous velocity was significantly decreased (p<0.001) following PRP in diabetic patients having proliferative retinopathy, as compared to the controls. In those having decrease in arterial velocity, the change was significant in PDR patients as compared to controls (p=0.006). There was increased arterial velocity observed with an average increase of 23% from the baseline but the difference was statistically significant (p=0.43).

Mendrinos et al.^18^ has commented that PRP causes vasoconstriction of retinal arterioles in the patients of proliferative retinopathy or severe nonproliferative retinopathy. This effect is consistent with the autoregulatory response expressed by the retinal blood vessels to increased inner retinal oxygen tension following PRP.

### Limitations of the study

We conducted a cross-sectional study, so some of the factors may differ among the different patients. Also, we didn’t collect the data regarding HbA1c and duration of diabetes.

## CONCLUSIONS

OCT angiography revealed there is significantly reduced optic nerve head blood flow in PRP treated eyes compared to non-PRP treated eyes.

### Recommendations

OCT Angiography can be used as a tool to measure optic nerve head blood flow.

### Author’s Contribution

**SAA:** Conception of idea, Data Collection.

**AAK:** Study Design, Manuscript writing, is responsible for integrity of study.

**HT:** Data Collection, Data Analysis.

**SS:** Manuscript writing.

## References

[1] Sasso FC, Pafundi PC, Gelso A, Bono V, Costagliola C, Marfella R (2019). Telemedicine for screening diabetic retinopathy:The NO BLIND Italian multicenter study. Diabetes Metabol Res Rev.

[2] Papatheodorou K, Banach M, Bekiari E, Rizzo M, Edmonds M (2018). Complications of diabetes 2017. J Diabetes Res.

[3] Aftab H, Ambreen A, Jamil M, Garred P, Petersen JH, Nielsen SD (2017). High prevalence of diabetes and anthropometric heterogeneity among tuberculosis patients in Pakistan. Trop Med Int Health.

[4] Hills AP, Arena R, Khunti K, Yajnik CS, Jayawardena R, Henry CJ (2018). Epidemiology and determinants of type 2 diabetes in south Asia. Lancet Diabetes Endocrinol.

[5] Gudla S, Tenneti D, Pande M, Tipparaju SM Diabetic retinopathy:pathogenesis, treatment, and complications. In book:Drug Delivery for the Retina and Posterior Segment Disease.

[6] Royle P, Mistry H, Auguste P, Shyangdan D, Freeman K, Lois N (2015). Pan-retinal photocoagulation and other forms of laser treatment and drug therapies for non-proliferative diabetic retinopathy:systematic review and economic evaluation. Health Technol Assess.

[7] Iwase T, Kobayashi M, Yamamoto K, Ra E, Terasaki H (2017). Effects of photocoagulation on ocular blood flow in patients with severe non-proliferative diabetic retinopathy. PLoS One.

[8] Budzynski E, Smith JH, Bryar P, Birol G, Linsenmeier RA (2008). Effects of photocoagulation on intraretinal PO2 in cat. Invest Ophthalmol Vis Sci.

[9] Regatieri CV, Branchini L, Carmody J, Fujimoto JG, Duker JS (2012). Choroidal thickness in patients with diabetic retinopathy analyzed by spectral-domain optical coherence tomography. Retina.

[10] Song Y, Tani T, Omae T, Ishibazawa A, Yoshioka T, Takahashi K (2018). Retinal blood flow reduction after panretinal photocoagulation in Type 2 diabetes mellitus:Doppler optical coherence tomography flow meter pilot study. PLoS One.

[11] Zhu Y, Zhang T, Wang K, Xu G, Huang X (2015). Changes in choroidal thickness after panretinal photocoagulation in patients with type 2 diabetes. Retina.

[12] Zhang Z, Meng X, Wu Z, Zou W, Zhang J, Zhu D (2015). Changes in choroidal thickness after panretinal photocoagulation for diabetic retinopathy:a 12-week longitudinal study. Invest Ophthalmol Vis Sci.

[13] Kang HM, Lee NE, Choi JH, Koh HJ, Lee SC (2018). Significant reduction of both peripapillary and subfoveal choroidal thickness after panretinal photocoagulation in patients with type 2 diabetes. Retina.

[14] Okamoto M, Matsuura T, Ogata N (2016). Effects of panretinal photocoagulation on choroidal thickness and choroidal blood flow in patients with severe nonproliferative diabetic retinopathy. Retina.

[15] Kim JT, Lee DH, Joe SG, Kim JG, Yoon YH (2013). Changes in choroidal thickness in relation to the severity of retinopathy and macular edema in type 2 diabetic patients. Invest Ophthalmol Vis Sci.

[16] Yamada Y, Suzuma K, Onizuka N, Uematsu M, Mohamed YH, Kitaoka T (2017). Evaluation of retinal blood flow before and after panretinal photocoagulation using pattern scan laser for diabetic retinopathy. Current Eye Res.

[17] Lee JC, Wong BJ, Tan O, Srinivas S, Sadda SR, Huang D (2013). Pilot study of Doppler optical coherence tomography of retinal blood flow following laser photocoagulation in poorly controlled diabetic patients. Invest Ophthalmol Vis Sci.

[18] Mendrinos E, Mangioris G, Papadopoulou DN, Dosso AA, Pournaras CJ (2010). Retinal vessel analyzer measurements of the effect of panretinal photocoagulation on the retinal arteriolar diameter in diabetic retinopathy. Retina.

